# Improved analysis of arabinoxylan-bound hydroxycinnamate conjugates in grass cell walls

**DOI:** 10.1186/s13068-020-01841-6

**Published:** 2020-12-10

**Authors:** Alexis Eugene, Catherine Lapierre, John Ralph

**Affiliations:** 1 Great Lakes Bioenergy Research Center, Wisconsin Energy Institute, University of Wisconsin, Madison, WI USA; 2 Institute Jean-Pierre Bourgin, INRAE, AgroParisTech, Université Paris-Saclay, Versailles, France; 3 Department of Biochemistry, University of Wisconsin, Madison, WI USA

**Keywords:** Ferulate, *p*-Coumarate, Acylation, Crosslinking, Dehydrodiferulates, Methanolysis

## Abstract

**Background:**

Arabinoxylan in grass cell walls is acylated to varying extents by ferulate and *p*-coumarate at the 5-hydroxy position of arabinosyl residues branching off the xylan backbone. Some of these hydroxycinnamate units may then become involved in cell wall radical coupling reactions, resulting in ether and other linkages amongst themselves or to monolignols or oligolignols, thereby crosslinking arabinoxylan chains with each other and/or with lignin polymers. This crosslinking is assumed to increase the strength of the cell wall, and impedes the utilization of grass biomass in natural and industrial processes. A method for quantifying the degree of acylation in various grass tissues is, therefore, essential. We sought to reduce the incidence of hydroxycinnamate ester hydrolysis in our recently introduced method by utilizing more anhydrous conditions.

**Results:**

The improved methanolysis method minimizes the undesirable ester-cleavage of arabinose from ferulate and *p*-coumarate esters, and from diferulate dehydrodimers, and produces more methanolysis vs. hydrolysis of xylan-arabinosides, improving the yields of the desired feruloylated and *p*-coumaroylated methyl arabinosides and their diferulate analogs. Free ferulate and *p*-coumarate produced by ester-cleavage were reduced by 78% and 68%, respectively, and 21% and 39% more feruloyl and *p*-coumaroyl methyl arabinosides were detected in the more anhydrous method. The new protocol resulted in an estimated 56% less combined diferulate isomers in which only one acylated arabinosyl unit remained, and 170% more combined diferulate isomers conjugated to two arabinosyl units.

**Conclusions:**

Overall, the new protocol for mild acidolysis of grass cell walls is both recovering more ferulate- and *p*-coumarate-arabinose conjugates from the arabinoxylan and cleaving less of them down to free ferulic acid, *p*-coumaric acid, and dehydrodiferulates with just one arabinosyl ester. This cleaner method, especially when coupled with the orthogonal method for measuring monolignol hydroxycinnamate conjugates that have been incorporated into lignin, provides an enhanced tool to measure the extent of crosslinking in grass arabinoxylan chains, assisting in identification of useful grasses for biomass applications.

## Background

Hydroxycinnamate conjugates in the cell wall have been the subject of increasing interest. *p*-Coumarate (*p*CA), especially, has been long known to acylate lignins and has been shown to arise on lignin polymers via polymerization of pre-formed monolignol *p*CA conjugates, along with the canonical monolignols [1–3]. Transferase genes have now been identified for this monolignol acylation via the activated intermediate *p*-coumaroyl-CoA [4–6]; the equivalent alternative viewpoint is that *p*CA (via *p*-coumaroyl-CoA) is esterified by monolignols. More recently, plants have been engineered to produce analogous monolignol ferulate (FA) conjugates [3, 7, 8], that also participate in lignification and, because ferulate is more compatible with the monolignols in its radical coupling reactions [2, 9], biosynthesize lignins with readily cleavable ester bonds in the lignin backbone, allowing more facile delignification of such materials [8, 10–12]. The DFRC (derivatization by reductive cleavage) method, which cleaves lignin β-ethers while leaving γ-esters intact, was extended to provide unambiguous evidence for the existence of such conjugates in lignin, and to assess their (relative) levels in the polymer [7, 13–17]; DFRC releases both *p*CA and FA conjugates (and in fact also benzoate, vanillate, and other conjugates) that are involved in β-ether units in lignin that can be cleaved by the method [13, 18–20]. The availability of the method lead to the discovery that various plants naturally use monolignol FA (ML-FA) conjugates in their lignification [15].

**Fig. 1:**
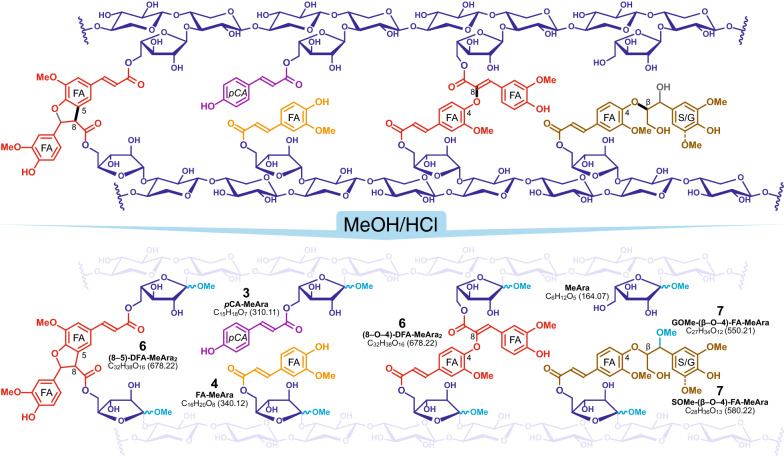
Methanolysis of hydroxycinnamoylated arabinoxylan. Top: Schematic arabinoxylan chains shown with the arabinosyl substitutions on every second unit as revealed recently [39], along with *p*CA and FA substitutions on arabinosyl units, two representative diferulates (8-*O*-4 and 8-5) formed by radical coupling, and a monolignol ferulate (ML-FA) conjugate, all known to be present in grass cell walls; acetate substitutions, also known, are not shown. The bonds produced by radical coupling are shown in bold-black, and the bonds formed by post-coupling rearomatization reactions (along with the OH from water addition in the case of the ML-FA coupling) are in gray. Bottom: The derived products released for structural analysis and quantification upon methanolysis that, ideally, methanolyzes the arabinosyl unit from the xylan backbone but leaves the arabinosyl esters intact. The original xylan chain is shown with low opacity for easier visualization of the source of the products, and the elemental formulas and exact mass (for MS analysis) are shown. As noted in the text, some actual hydrolysis can occur (producing acids) and some methanolysis of the hydroxycinnamate-arabinosyl ester can occur, producing DFAs with only a single Ara unit; the improved method here produces lower amounts of these undesirable side-products and more of the desired *p*CA-MeAra **3**, FA-MeAra **4**, and the various DFA-diMeAra isomers **6** (as well as ML-FA-MeAra cross-products **7**); the compound numbers are from Fig. 2

FA and *p*CA esters have also long been known on arabinoxylans in grasses, and commelinid monocots in general [2, 21–29], Fig. 1. Both, but particularly FA-polysaccharide esters, have been demonstrated to be involved in radical coupling reactions to cross-link cell wall polysaccharides, both with other hydroxycinnamate-bearing polysaccharide chains and with lignin. The genes/enzymes involved in arabinoxylan acylation have been more difficult to elucidate and the actual nature of the acceptor (poly)saccharide moiety remains elusive; a gene for *p*-coumaroylation is reasonably certain [30, 31], but the ferulate analog remains putative [32].

Having orthogonal analytical methods to delineate between hydroxycinnamates that acylate monolignols vs. polysaccharides is particularly beneficial for research in this area. We recently introduced a method to clip arabinosyl units from arabinoxylan polysaccharides releasing hydroxycinnamoylated methyl arabinosides for analysis [33], Fig. 1. The 1-*O*-methyl arabinosides vs. the parent (1-OH) arabinosides themselves, allow for improved HPLC producing sharper chromatograms than the original method using aqueous HCl [34], presumably because the α- and β-anomers of the parent arabinose could interconvert during chromatography, whereas the methyl arabinosides are stable. The major species of interest identified with this method are methyl 5-*O*-feruloyl arabinofuranoside (FA-MeAra **4**) and methyl 5-*O*-*p*-coumaroyl arabinofuranoside (*p*CA-MeAra **3**), as well as various dehydrodiferulates (DFA) **6** and ferulate-monolignol cross-products **7** in which the ferulate moieties are esterified as 1-*O*-methyl arabinosides [33, 34], Fig. 1.

The mild acidolysis procedure provides a simple, straightforward way of determining the relative acylation of grass arabinoxylan, but we came to realize that the presence of ~ 10% water in the acidolysis reagent leads to some undesirable hydrolysis that cleaves the esters and results in small amounts of arabinose rather than 1-*O*-methyl-arabinofuranoside units. As a result, some monomeric free hydroxycinnamic acids are produced (where as free acids are essentially not present in the cell wall) along with a selection of mono-methyl-arabinosylated diferulates that complicate the chromatogram. Here, we describe an improved ‘anhydrous’ method that limits such hydrolytic reactions, while still being able to cleave the glycoside linking arabinose units to the xylan backbone, Fig. 1.

## Results and discussion

As described in “Methods” section, the new procedure utilizes anhydrous methanolic HCl (with dioxane as a co-solvent) to limit aqueous hydrolysis of both the hydroxycinnamate ester and the arabinosyl unit from the xylan backbone. The methanolic-HCl reagent is conveniently prepared from commercially available anhydrous methanol (and dioxane) by carefully adding acetyl chloride in the simple method described by Fieser and Fieser [35]. We admit that the conditions are not strictly anhydrous as the biomass sample comes with its own moisture and exchangeable hydroxyls. We were originally not certain that such conditions would continue to efficiently clip off the arabinose units, fearing that some water might be necessary; that trepidation appeared to be groundless, however, as methanolysis was found to be essentially as facile as hydrolysis. We tested whether yields could be improved by adding the acetyl chloride directly to samples already suspended in anhydrous methanol/dioxane; however, as the FA-MeAra **4** yield only increased by about 2%, we contend that the simplicity of using the pre-prepared hydrolysis solution outweighs any slight yield benefit that might be realized.

**Fig. 2:**
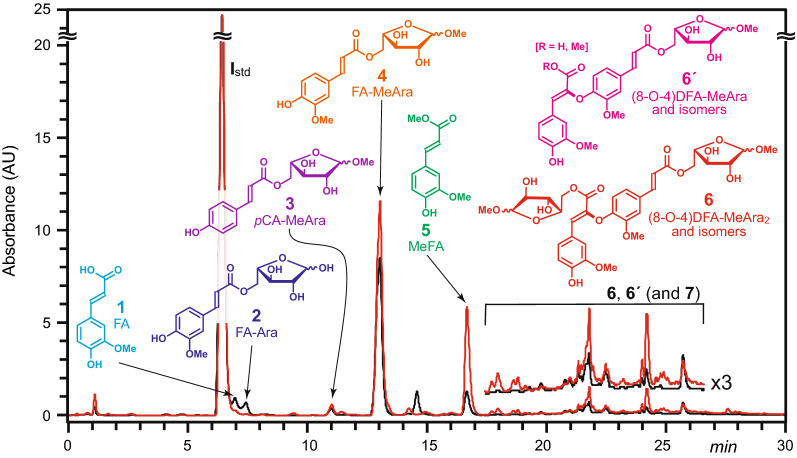
UV–vis chromatograms of maize IDF after mild acidolysis. Black trace was processed using the original acidolysis method; red trace was the new anhydrous method described herein. Internal standard, *I*_std_; ferulate **1**; FA-Ara **2** (unmethylated, *m*/*z* 325); *p*CA-MeAra **3**, FA-MeAra **4**; MeFA, methyl ferulate, **5**; DFAs **6** (one isomer, the 8-O-4-DFA, is shown) with two or one (**6ʹ**) MeAra attached, also shown vertically expanded ×3; monolignol-FA-MeAra coupling products **7** (Fig. 1) are also in this region

Figure 2 shows the UV chromatograms for a sample of maize insoluble dietary fiber (IDF) that underwent both the normal (black) and anhydrous (red) acidolysis processes. The main products of mild acidolysis are marked **1**–**7**. Peak **1** is free FA, formed from the hydrolysis of arabinose-bound ferulate. This peak is significantly decreased when mild acidolysis is performed under the more anhydrous conditions, indicating that less hydrolysis of FA-MeAra is occurring. *p*-Coumaric acid (*p*CA, eluting at 4.8 min) is also reduced, although the peak is too small to see in this chromatogram. Peak **2** appears only in the original method and is due to unmethylated FA-Ara (*m*/*z* 325); similar small peaks occur throughout the chromatogram for the (partially) unmethylated analogs of all the possible products when aqueous HCl is used. Performing the acidolysis in the near-absence of water clearly aids in the methanolysis (rather than the competing hydrolysis) of the arabinosyl units, simplifying the array of products formed. Peaks **3** and **4** are *p*CA-MeAra and FA-MeAra, typically the most abundant desired products from mild acidolysis of grasses. The peak at 14.5 min (with an apparent mass of 472, that is more abundant in the older method) remains unidentified at this time. Peak **5** is due to methyl ferulate. The elevation of peak **5** (and also methyl *p*-coumarate, not shown) suggests that more ferulate is clipped from its arabinose unit but peak **4** is also enhanced under the more anhydrous acidolysis conditions, so the method remains an improvement for the methyl-arabinosylated compounds of most interest. Finally, the grouped region in Fig. 2 is where a series of different DFAs **6** conjugated to either two or one (denoted as **6ʹ**) methyl arabinoside units elutes. Monolignol ferulate (ML-FA) cross-products **7**, Fig. 1, are also found in this region. The presence of these species is direct evidence of the crosslinking nature of arabinose-bound ferulate. It is clear from the UV chromatograms that there are again more of these products recovered with the dry method (red chromatogram).

**Fig. 3:**
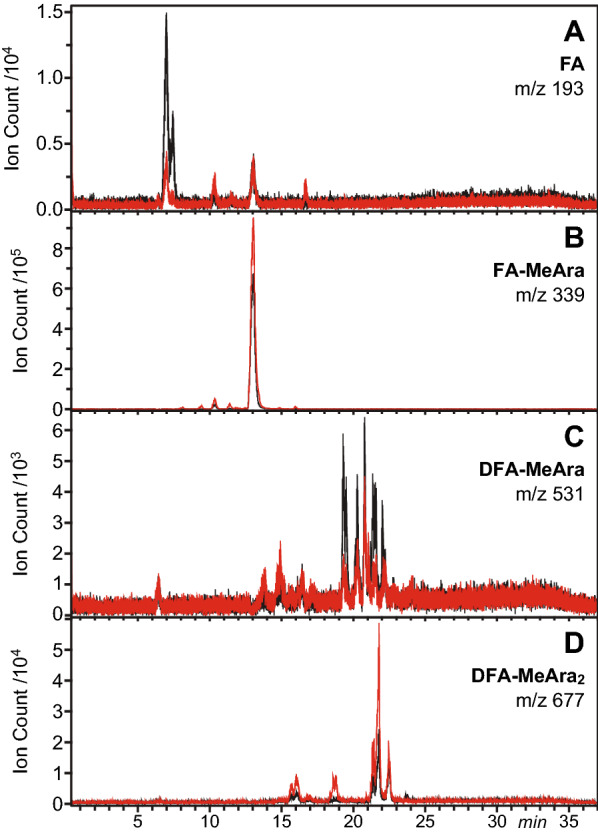
Extracted-ion chromatograms. Extracted-ion chromatograms (EIC) are shown for *m*/*z* 193, 339, 531, and 677 from the samples in Fig. 2. Black trace was processed using the original acidolysis method; red trace was the new anhydrous method. **a**, **c** Show that levels of the hydrolyzed products FA and DFA-MeAra are reduced under the drier acidolysis conditions. **b**, **d** Show that the unhydrolyzed esters with fully methanolyzed Ara units, FA-MeAra **4** and DFA-MeAra_2_ **6**, are enhanced in the anhydrous method

**Fig. 4:**
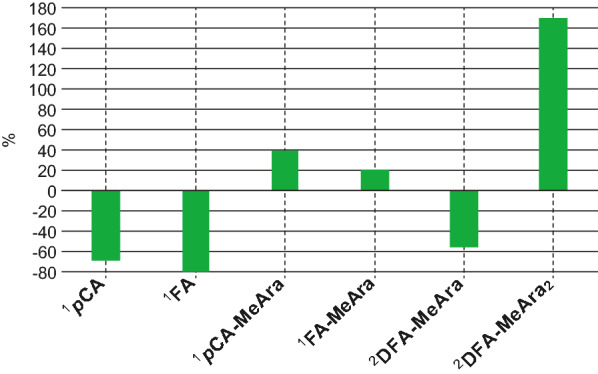
Yield comparison for various undesired and desired products. Percent differences in yields between anhydrous acidolysis and the original method [33]. ^1^Based on authentic standards. ^2^No standard available; calculated directly from peak areas in EIC mass spectra

Figure 3 shows the key differences between wet and anhydrous acidolysis in more detail. Panel a displays the extracted-ion chromatogram (EIC) for *m*/*z* 193 from the samples in Fig. 2, in which *Z*- and *E*-ferulic acid **1** elute at 7.0 and 7.5 min; both are reduced (as noted above) indicating that less ester hydrolysis has taken place. Panel b is the EIC for *m*/*z* 339, corresponding to FA-MeAra **4**, which elutes at 13.1 min; the level of this crucial marker compound (for ferulates on arabinoxylan) is elevated. Similarly, panels c and d represent related compounds at various stages of hydrolysis. Figure 3c presents the EIC for DFA-MeAra **6ʹ** at *m*/*z* 531; all of the mono-MeAra DFAs are reduced, again indicating that less arabinose hydrolysis has occurred. Finally, panel d shows the EIC for *m*/*z* 677, which is DFA-MeAra_2_ **6**; these crucial peaks are all substantially elevated, again because less ester hydrolysis, and more methanolysis and less hydrolysis of the arabinosyl glycosidic position, has occurred. There are many different isomers of DFA arabinosides **6** possible as identified and discussed previously [33], accounting for the plethora of peaks in this chromatogram. An estimate of the yield changes from using anhydrous conditions for acidolysis is presented in Fig. 4, clearly showing how the peaks of interest are enhanced over those resulting from hydrolysis.

The aim of this paper was to present the improved method for the analysis of hydroxycinnamates, monomeric and dimeric, on grass arabinoxylans. The method minimizes the undesirable ester-cleavage of arabinose from ferulate and *p*-coumarate esters, and from diferulate dehydrodimers, and produces more methanolysis vs. hydrolysis of xylan-arabinosides, improving the yields of the desired feruloylated and *p*-coumaroylated methyl arabinosides and their diferulate analogs. Free ferulate and *p*-coumarate produced by ester-cleavage were reduced by 78% and 68%, respectively, and 21% and 39% more feruloyl and *p*-coumaroyl methyl arabinosides (**4** and **3**) were detected in the more anhydrous method. The new protocol resulted in an estimated 56% less combined diferulate isomers **6ʹ** in which only one acylated arabinosyl unit remained, and 170% more combined diferulate isomers **6** conjugated to two arabinosyl units. The results of quantifying the monomeric methyl hydroxycinnamoyl arabinosides **3** and **4** from a few grass samples, using the new method, are given in Table 1. This protocol will be used in our laboratories from this point on and is recommended to other research groups pursuing such analyses.

**Table 1:** Amounts of methyl hydroxycinnamoyl arabinosides released from selected monocot samples

Sample	*p*CA-MeAra 3 (mg/g)	FA-MeAra 4 (mg/g)
Sorghum	2.51 ± 0.57	11.22 ± 0.73
Switchgrass	7.83 ± 0.39	8.96 ± 0.41
Maize IDF	3.62 ± 0.26	33.10 ± 1.71
Rye IDF	0.81 ± 0.05	19.08 ± 1.06

Analyzed using the improved method described in “Methods” section. Values are means of duplicate runs ± the standard deviation

## Conclusions

Overall, mild acidolysis is a powerful tool for analyzing the relative acylation of grass arabinoxylans by *p*CA and FA. Although not all peaks resulting from either hydrolysis of the ester or of the arabinosyl unit from the xylan were eliminated, their levels were significantly reduced. For the analysis of polysaccharide-bound *p*CA and FA, the levels of the methyl arabinosides **3** and **4** were elevated. In addition, for the DFAs that are most important for evaluation of the extent of polysaccharide crosslinking in grasses, the mono-methyl-arabinosylated DFAs (DFA-MeAra **6ʹ**) were reduced, and the fully di-methyl-arabinosylated DFAs (DFA-MeAra_2_ **6**) were significantly elevated. Because of the higher yields of desirable products and the simplification of the chromatograms from the significant elimination of hydrolysis (vs. methanolysis) products, we highly recommend this method for determining the (relative) levels of hydroxycinnamates and their dimers (and monolignol cross-products) on arabinoxylans, and for its use with the complimentary DFRC-based method to independently analyze lignin-bound hydroxycinnamates.

## Methods

### Chemicals

ACS grade dioxane (Sigma-Aldrich) was distilled immediately prior to use. Anhydrous methanol (Alfa Aesar, 99.9%, packed under argon with septum) was used as is. Aqueous 2 M HCl was made from concentrated HCl (Sigma-Aldrich, 37%) in ultrapure water (18.2 MΩ cm^−1^, Thermoscientific E-pure). Acetyl chloride (98%) was from Sigma-Aldrich. FA (Aldrich, 99%), *p*CA (Sigma, 98%), and *m*-coumaric acid (Acros, 99%) were used as is. FA-MeAra, and *p*CA-MeAra were synthesized as previously described [33, 36, 37]. Chromatography was performed with LC–MS grade 0.1% formic acid and acetonitrile obtained from Fisher Scientific.

### Plant materials

Insoluble dietary fiber (IDF) from maize and rye was obtained as described previously [38]. Briefly, maize or rye bran was ground to < 0.5 mm, defatted with *n*-hexane, and treated with α-amylase. The residue was extracted several times with water and ethanol, and freeze dried for 48 h. Energy sorghum and switchgrass were supplied by the Great Lakes Bioenergy Research Center’s material production chain from the 2019 harvest year. Whole-cell-wall samples were pre-ground to < 0.5 mm and then extracted three times with water and ethanol, and once with acetone before being freeze dried for 48 h; the product is generally termed the ‘cell wall residue’ or CWR, or in the food arena as IDF. The dried extract-free IDF material was then ball milled.

### Mild acidolysis

Aqueous mild acidolysis (‘normal acidolysis’) was performed as described previously [33]. In a glass vial (Chemglass Life Sciences, 2 dram), 10 mg of extract-free plant material was mixed into 1 mL of aqueous acidolysis reagent consisting of dioxane, methanol, and 2 M HCl_(aq)_ in a 60/30/10 (v/v) ratio. The vial was capped and heated at 80 °C for 3 h with stirring. Anhydrous mild acidolysis was performed as described above except the samples were incubated with 1 mL of a dry acidolysis reagent. This reagent was prepared by carefully adding 0.7 mL of acetyl chloride to 20 mL of 70/30 (v/v) freshly distilled dioxane/absolute methanol, following the Fieser and Fieser method for making anhydrous methanolic HCl [35]. As acetyl chloride is moisture sensitive, the dioxane and methanol were kept as dry as possible (using only syringe extraction through the septum), to prevent hydrolysis. After reaction, 100 µL of 30 mM *m*-coumaric acid was added as internal standard. Then, ~ 2 mL of water was added and the mixture was extracted with three portions of ethyl acetate. The organic layers were combined and dried over anhydrous magnesium sulfate, gravity-filtered (VWR 415 filter paper), and the solvent evaporated to dryness on a rotavap. The residue was dissolved in 1.0 mL of acetonitrile and syringe-filtered (0.2 µm) before liquid chromatography–mass spectrometric (LC–MS) analysis.

### LC–MS analysis

The samples were analyzed on a Shimadzu Nexera X-2 UHPLC equipped with a Phenomenex XB-C18 column (1.7 µm, 100 × 2.1 mm) and a 2.0 µL injection volume. Gradient elution was performed using solvents *A* = 0.1% formic acid and *B* = acetonitrile. The gradient program was as follows: 0–2 min, 5% *B*; ramp to 18% *B* at 17.5 min; ramp to 50% *B* at 32 min; hold for 1 min. Detection was performed with a Shimadzu SPD M30A photodiode array and a Bruker Impact II Ultra-high Resolution QqTOF mass spectrometer operating in negative-ion mode electrospray ionization. UV spectra were recorded in the 250–400 nm range and the MS was operated with capillary voltage, 3.5 kV; nebulizing gas pressure, 4.0 bar; drying gas temperature, 210 °C; drying gas flow, 8 L min^−1^, *m*/*z* range, 50–1000. FA (Aldrich, 99%), *p*CA (Sigma, 98%), FA-MeAra **4**, and *p*CA-MeAra **3** were quantified in the MS using authentic standards with *m*-coumaric acid (Acros, 99%) as the internal standard; the arabinose-containing standards were the same as those used in the original method [33], and their synthesis has been described [36, 37]. Standards were not available for DFA-MeAra and DFA-MeAra_2_, so changes in these were estimated from a simple comparison of MS peak areas.

## Data Availability

The datasets used and/or analyzed during the current study are available from the corresponding author on reasonable request.

## Abbreviations

FA: Ferulic acid **1**
*p*CA: *p*-Coumaric acid
FA-Ara: 5-*O*-Feruloyl arabinofuranoside **2**
FA-MeAra: Methyl 5-*O*-feruloyl arabinofuranoside **4**
*p*CA-MeAra: Methyl 5-*O*-*p*-coumaroyl arabinofuranoside **3**
DFA: Dehydrodiferulate or (dehydro)diferulic acid
DFA-MeAra: Methyl dehydrodiferuloyl monoarabinofuranoside **6ʹ**
DFA-MeAra_2_: Dimethyl dehydrodiferuloyl diarabinofuranoside **6**
EIC: Extracted-ion chromatogram
IDF: Insoluble dietary fiber (≈ CWR, cell wall residue)
LC–MS: Liquid chromatography–mass spectrometry (or spectrometer
